# Transition from inflammation to proliferation: a critical step during wound healing

**DOI:** 10.1007/s00018-016-2268-0

**Published:** 2016-05-14

**Authors:** Ning Xu Landén, Dongqing Li, Mona Ståhle

**Affiliations:** 1 Unit of Dermatology and Venereology, Molecular Dermatology Research Group, Department of Medicine, Center for Molecular Medicine (CMM), L8:02, Karolinska Institutet, SE-171 76 Stockholm, Sweden; 2 Unit of Dermatology and Venereology, Karolinska University Hospital, Solna, Sweden

**Keywords:** Macrophage, Fibroblast, Bioactive lipid mediator, Reactive oxygen species, Toll-like receptor, Transcription factor, MicroRNA

## Abstract

The ability to rapidly restore the integrity of a broken skin barrier is critical and is the ultimate goal of therapies for hard-to-heal-ulcers. Unfortunately effective treatments to enhance healing and reduce scarring are still lacking. A deeper understanding of the physiology of normal repair and of the pathology of delayed healing is a prerequisite for the development of more effective therapeutic interventions. Transition from the inflammatory to the proliferative phase is a key step during healing and accumulating evidence associates a compromised transition with wound healing disorders. Thus, targeting factors that impact this phase transition may offer a rationale for therapeutic development. This review summarizes mechanisms regulating the inflammation–proliferation transition at cellular and molecular levels. We propose that identification of such mechanisms will reveal promising targets for development of more effective therapies.

## Introduction

Skin is the main barrier protecting us from the often hostile environment. Upon injury, rapid closure of the wound and prompt regeneration of the damaged skin are critical to restore barrier function. Effective repair requires communication and interplay between many different cell types and this process is precisely orchestrated and regulated at multiple levels [1]. The wound healing process is usually characterized as four sequential but overlapping phases: haemostasis (0–several hours after injury), inflammation (1–3 days), proliferation (4–21 days) and remodelling (21 days–1 year) [1]. Deregulation of any of these steps results in impaired healing, e.g., chronic hard-to-heal ulcers or excessive scarring, which presents a major and increasing health and economic burden to our society [2, 3]. Current treatments for impaired wound healing focus mainly on optimisation of controllable healing factors, e.g., clearance of infection, mechanical protection and nutritional support. Few targeted approaches have been developed to date, including mainly topical application of growth factors, unfortunately with limited clinical efficacy [4]. Identification of new therapeutic targets and development of more effective treatments are needed.

Transition from the inflammatory to the proliferative phase represents a key step during wound healing. The inflammatory phase is essential leading to haemostasis and recruitment of the innate immune system, which defends us against the attack of invading pathogens and help remove dead tissues [1]. However, prolonged inflammation is detrimental and may result in deregulated differentiation and activation of keratinocytes, impeding the progress through the normal stages of wound healing [2]. Severe inflammation has also been associated with excessive scarring [5]. Compared with the process of initiation and amplification of the inflammatory response, we know much less about how inflammation is resolved during normal wound healing, which is prerequisite for understanding the pathogenesis of persistent inflammation in chronic wounds [6]. Moreover, the next step, proliferation, is tightly connected with the inflammatory response, and also plays an important role in resolving inflammation. This review will focus on the inflammation-proliferation transition in normal physiologic as well as in impaired wound healing and highlight factors that can regulate this process at cellular and molecular levels.

## Physiologic skin wound healing

Wound healing is often described as a drama, with the interplay of a multitude of different cell types, and is precisely directed to serve the ultimate goal: prompt healing [1]. Although many questions remain, extensive studies in the field have provided a general picture about this fundamental biological process.

### Haemostasis phase

Once the skin gets injured, exposure of collagen initiates the intrinsic and extrinsic clotting cascades. Thrombocytes aggregate and trigger vasoconstriction to reduce blood loss, which results in hypoxia, increased glycolysis and pH changes [7, 8]. A blood clot is formed to fill up the wound bed, which serves as a provisional wound matrix, providing a scaffold for the migration of different cell players. After a 5- to 10-min vasoconstriction, blood vessels are dilated, thrombocytes and leukocytes migrate into the provisional matrix [1]. Degranulation of platelets activates the complement cascade, which stimulates inflammatory cells and kills bacteria [9]. Moreover, a variety of cytokines and growth factors are released into the wound during this stage, mediating the communication and synergizing the activity of different cell players to accomplish the task of healing. A classic view of cytokines and growth factors in skin wound healing was summarized elsewhere [10].

### Inflammation phase

**Fig. 1:**
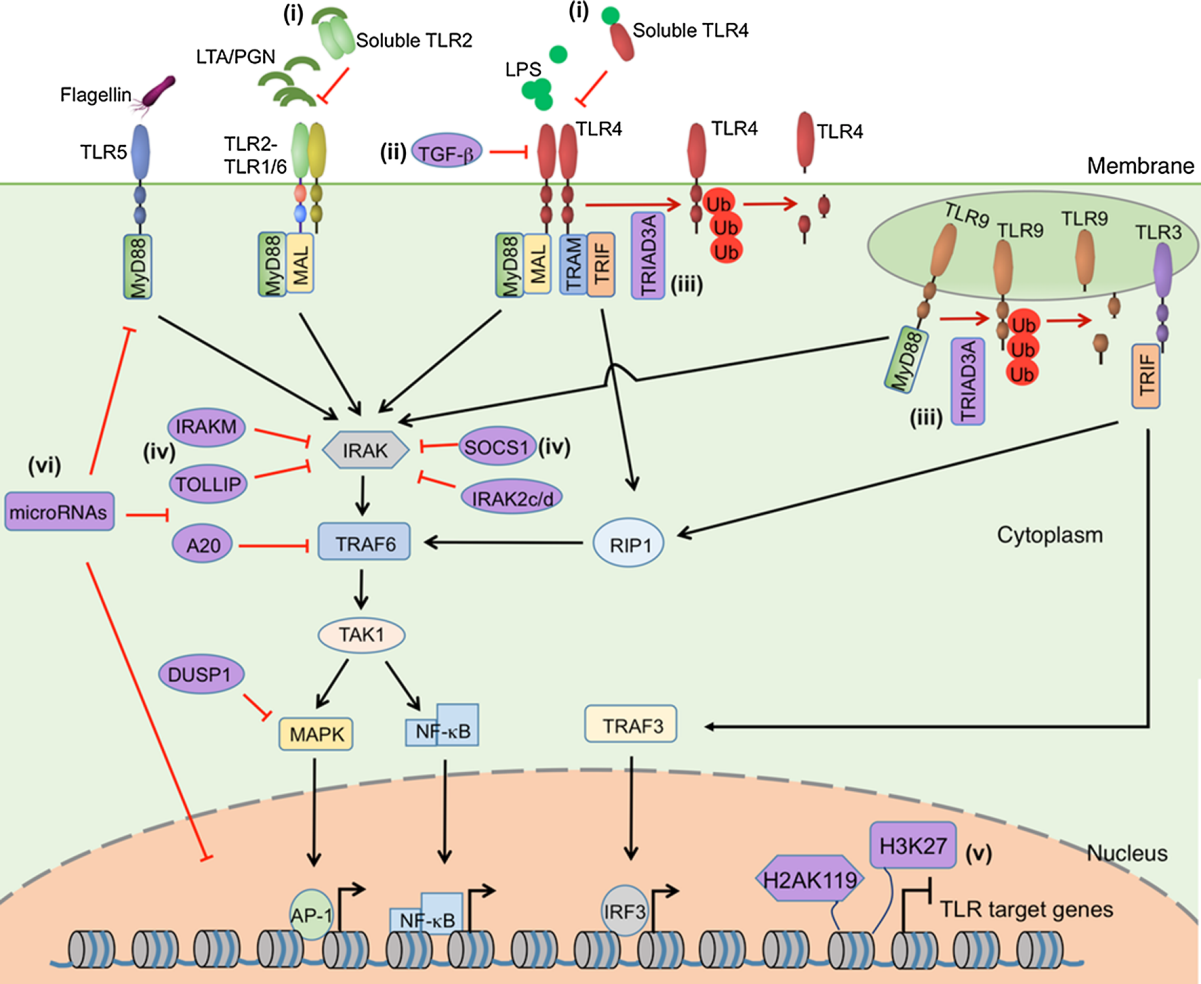
Negative regulators of Toll-like receptor signaling. TLR2, TLR4, TLR5, TLR9 activate myeloid differentiation primary-response protein 88 (MyD88)-dependent pathway. MYD88-adaptor-like protein (Mal) recruits MyD88 to TLR2 and TLR4. TLR3 activates TIR-domain-containing adapter-inducing interferon-β (TRIF)-dependent pathway. In association with TRIF-related adaptor molecule (TRAM), TLR4 can also activate TRIF-dependent pathway. Upon stimulation, MyD88 recruits IL-1 receptor-associated kinase (IRAK), which is activated by phosphorylation and then associates with TNF receptor-associated factor 6 (TRAF6), leading to the activation of transforming growth factor β-activated kinase (TAK1). TAK1 further activates the transcription factors NF-κB and AP-1 through MAPK and NF-κB pathways, respectively. TRIF binds to receptor-interacting protein 1 (RIP1) and TRAF6, also leading to the activation of NF-κB and MAPKs. TRIF also activates interferon regulatory factor 3 (IRF3) through TNFR-associated factor 3 (TRAF3). A plethora of inhibitory mechanisms have been identified in TLR signaling:* (i)* interference of ligand binding, e.g., soluble forms of TLR2 and TLR4 compete with membrane-bond forms of TLRs for ligands binding;* (ii)* reduction of TLR expression, e.g., TGF-β suppresses the expression and function of TLR4;* (iii)* degradation of TLRs, e.g., TRIAD3A binds to the cytoplasmic domain of TLR4 and TLR9 and promotes their ubiquitylation and degradation;* (iv)* inhibition of TLR downstream signaling, e.g., SOCS1, IRAKM, TOLLIP, IRAK2c/d, A20 and DUSP1;* (v)* change of structures of target genes through chromatin remodeling and histone modification, e.g., H2AK119 ubiquitylation and H3K27 trimethylation inhibit the expression of TLR-signal-targeted genes;* (vi)* microRNAs can regulate TLR signaling by targeting TLRs, downstream signaling proteins, related regulatory molecules, transcription factors as well as genes induced by TLR signaling. The figure was made with tools from www.proteinlounge.com

In the inflammatory phase, involving mainly activation of the innate immune system, neutrophils and monocytes rapidly migrate into the injured skin. This phase is actually concurrent with haemostasis, and described as the early stage of wound healing [11]. As a consequence of injury, resident skin cells, e.g., keratinocytes, macrophages, dendritic cells and mast cells, are exposed to danger signals, which in general can be divided into two categories: (a) damage-associate molecular patterns (DAMPs), i.e., molecules released by stress cells undergoing necrosis, such as the intracellular proteins, DNA and RNA; (b) pathogen-associated molecular patterns (PAMPs), which are pathogen-specific molecules not found in the host, e.g., bacterial essential polysaccharides and polynucleotides [12]. Upon skin injury these danger signals are recognized by pattern recognition receptors, among which toll-like receptors (TLRs) are the best characterized ones. TLRs are constitutively expressed on host cells and stimulation of TLRs induces the activation of intracellular signalling pathways, including the nuclear factor kappa-light-chain-enhancer of activated B cells (NF-κB) and mitogen-activated protein kinases (MAPK) pathways, which lead to the expression of a large number of genes, including cytokines, chemokines and antimicrobial peptides, to initiate and perpetuate the inflammatory response (Fig. 1) [13, 14].

In response to chemokines, complement and by-products of bacterial degradation, neutrophils are recruited from the circulation to the wound site in the early inflammatory stage in a multistep process [1, 9]. Chemokines induce the expression of adhesion molecules, e.g., intercellular adhesion molecule 1 (ICAM1), vascular cell adhesion molecule 1 (VCAM1) and e-selectin (SELE), on endothelial cells, which mediate the adherence of neutrophils to the wall of blood vessels [15]. Adherence to endothelial cells and exposure to chemokines change the cytoskeleton of neutrophils, which lead to neutrophil extravasation [15]. Once outside the blood vessel, neutrophils are exposed to a chemokine gradient within the skin and migrates towards the higher concentration, the site where these chemokines are released, i.e., the wound site. It is noteworthy that all leucocytes, not only neutrophils, use this mechanism of localisation [15]. If the wound does not get infected, neutrophils usually remain for 2–5 days [16]. They perform phagocytosis to remove pathogens and cell debris [16]. After being engulfed into the phagosome of the neutrophil, the microbe is killed and digested by toxic cationic enzymes and oxygen metabolites [16]. Neutrophils also produce cytokines, e.g., tumor necrosis factor (TNF)-α, interleukin (IL)-1β and IL-6, to amplify the inflammatory response [11]. Moreover, they exert debridement by releasing a variety of antimicrobial substances, e.g., cationic peptides and proteinases [11].

**Fig. 2:**
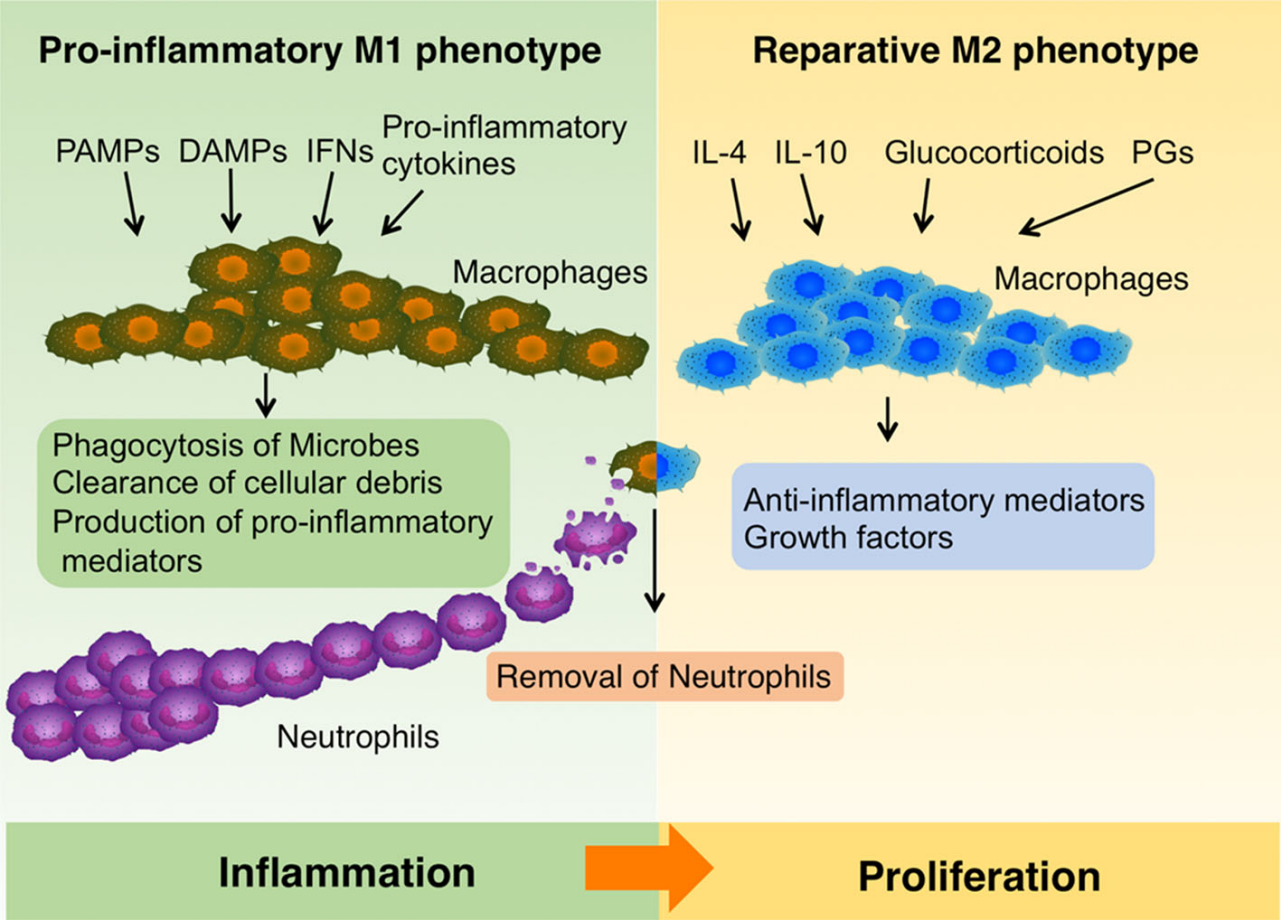
The roles of macrophage in wound healing. In the early phase of wound repair, upon exposure to pro-inflammatory cytokines, interferons (IFNs), PAMPs or DAMPs, infiltrating monocytes and resident macrophages are activated and mainly acquire a pro-inflammatory M1 phenotype. They perform phagocytosis of microbes, scavenge cellular debris and produce pro-inflammatory mediators. Later during healing process, IL4, IL-10, Glucocorticoids, Prostaglandins (PGs) and modulators of glucose and lipid metabolism induce macrophages to transit to a reparative M2 phenotype, which secret anti-inflammatory mediators and growth factors. Macrophages also remove neutrophils in the wounds by phagocytosis, a central element to induce the M1-M2 phenotype switch of macrophages. The figure was made with tools from www.proteinlounge.com

Approximately 3 days after injury, monocytes are recruited to the injury site, where they differentiate into macrophages and support healing (Fig. 2). Macrophages are important players facilitating the inflammatory–proliferative phase transition during wound healing, and will be discussed in a separate section.

In normal skin wound healing, the inflammation usually lasts for 2–5 days and ceases once the harmful stimuli have been removed; even though the immune responses continue through the whole procedure of wound healing, evolving through progressive states of specific leukocyte involvement and function (reviewed in [12]). The adaptive immune system, the other arm of immunity, provides a more delayed but specific response carried out by B and T cells. B cells not only secret antibodies, but also impact immune response by production of various cytokines and growth factors, antigen presentation, regulation of T cell activation and differentiation, and regulation of lymphoid organization [17]. B cell has been shown to present in wound tissue [18] and play a critical role in healing [19]. In wound repair, T lymphocytes function as growth factor-producing cells as well as immunological effector cells [20]. Specific deficiency of CD4 or CD8 lymphocytes changes the infiltration of inflammatory cells and the profiles of cytokine expression in skin wounds, while does not impair wound closure in mouse [21]. A prolonged and increased presence of T cells and a changed CD4-CD8 ratio have been observed in human chronic wounds [22]. Foxp3-expressing regulatory T cells (Tregs) are a dynamic and heterogeneous population of cells that control immune responses and prevent autoimmunity. There are a large number of Tregs presenting in the skin [23]. Recent studies show that activated Tregs accumulate in skin wound, which attenuate interferon (IFN)-γ production and proinflammatory macrophage accumulation, facilitating wound repair through epidermal growth factor receptor (EGFR) pathway [24].

Recently, several cell types, which bridge between innate and adaptive immunity, have been shown to play key roles in skin wound healing. Upon injury, plasmacytoid dendritic cells (pDCs) infiltrate in skin wounds at the same time as neutrophils [25]. pDCs sense host-derived nucleic acids released in the wound and transiently produce type I interferons (IFN-α/β) via TLR7- and TLR9-dependent mechanisms, which process is critical for the induction of early inflammatory responses and re-epithelialization of injured skin [25]. Langerhans cells (LCs) are a specialized subset of epidermal dendritic cells, which serve as first-line defender, contributing to epidermal immune surveillance. Increased epidermal LCs has been observed at wound edges during early phases of normal wound healing, although the exact protective mechanism of these cells is unknown [26, 27]. Moreover, higher number of LCs in the epidermis of diabetic foot ulcers has been reported to correlate with healing outcome [27]. Different from the well-defined αβT cell, γδT cell is a subset of T cells expressing T cell antigen recognition receptor (TCR) composed of γ and δ subunits. The subpopulation of γδT cells in the epidermis is known as dendritic epidermal T cells (DETC) [12]. In skin wounds, γδT cells can recognize and eliminate damaged keratinocyte, release growth factors, e.g., fibroblast growth factor (FGF)-7, keratinocyte growth factor (KGF)-1 and insulin-like growth factor (IGF)-1, which stimulate proliferation of neighbouring healthy keratinocytes (reviewed in [12]). In human acute wounds both αβ- and γδ- skin-resident T cells have been shown to actively produce IGF-1, whereas skin-resident T cells isolated from chronic wounds do not express IGF-1 and exhibit an unresponsive state [28]. Also, a subpopulation of γδT cells produces IFN-γ, enhancing the antimicrobial, antitumor and other functions of NK and αβT cells. Another subpopulation of γδT cells produce IL-17 and induce expression of multiple host-defense molecules in epidermal keratinocytes, promoting wound healing [29].

The immune system plays an active role not only in the inflammatory phase, but also throughout the whole wound healing process. Compared with innate immunity, our knowledge regarding the role of adaptive immunity in wound healing is sparse. Understanding the delicate immunologic balance is an important task for research on wound healing. This review will primarily focus on the role of innate immunity in relation to inflammation.

### Proliferation phase

As the inflammation subsides, proliferation becomes a major theme with the focus on covering the wound surface (i.e., re-epithelialization), restoring the vascular network and forming granulation tissue.

Re-epithelialization requires migration and proliferation of keratinocytes. In a few hours to 1 day after injury, the existing wound-edge keratinocytes start to migrate. To generate more cells to cover the wound, keratinocytes at the basal layer of the wound edge and epithelia stem cells from nearby hair follicles or sweat glands start proliferating approximately 2–3 days after injury [30]. Migration is triggered by loss of contact inhibition and physical tension at cell adhesion structures, i.e., desmosomes and hemidesmosomes, which activates membrane-associated kinases, thus leading to increased membrane permeability for calcium. This is a signal for reorganization of cytoskeleton driving migration. Meanwhile, the migrating cells are released from their original sites by collagenase and elastase. Details for this process were summarized elsewhere [31]. Migration stops when the cells get in contact and new adhesion structures are formed. Keratinocytes secrete proteins to rebuild the basement membrane [31]. Re-epithelialization can be stimulated by a variety of wound–related signals, e.g., nitric oxide, which is mainly synthesized by macrophages [32], cytokines and growth factors, including epidermal growth factor (EGF), KGF, IGF-1, and nerve growth factor (NGF), secreted from multiple cell types in the wounds [10].

Restoring the network of blood vessels is important, since nutrients and oxygen are needed during wound repair. The process of new blood vessel formation, also known as ‘angiogenesis’, is initiated by growth factors, e.g., vascular endothelial growth factor (VEGF), platelet-derived growth factor (PDGF), basic fibroblast growth factor (bFGF), and the serine protease thrombin in the wounds, which activate the endothelial cells of existing vessels [33]. After secreting proteolytic enzymes to dissolve the basal lamina, the endothelial cells escape from the existing vessels, proliferate and migrate towards the source of the angiogenic stimulus [33]. These sprouts form vessel lumen, differentiate into arteries and venules and mature by recruitment of pericytes and smooth muscle cells [33]. In addition, bone marrow-derived endothelial progenitors can also form vessels *de novo*, a process known as vasculogenesis, [11, 34].

In the proliferation phase, the provisional wound matrix formed during haemostasis is replaced by granulation tissue, consisting of a large amount of fibroblasts, granulocytes, macrophages, blood vessels, in complex with collagen bundles, which partially recovers the structure and function of the wounded skin [35]. Fibroblasts play a central role in the formation of the granulation tissue, which migrate mainly from the nearby dermis to the wound in response to cytokines and growth factors, e.g., PDGF, transforming growth factor (TGF)-β and bFGF, produced by platelets and macrophages in the wounds [35, 36]. If the wound condition is long lasting, fibroblasts in the wounds may also originate from fibrocytes, which is a group of bone marrow-derived mesenchymal progenitor cells [37, 38]. Circulating fibrocytes migrate to regions of skin injury and promote healing not only by contributing to a subset of fibroblasts in the wounds, but also by producing cytokines, chemokines, and growth factors, serving as antigen presenting cells as well as enhancing angiogenesis [39]. After migrating into the provisional wound matrix, fibroblasts proliferate and produce proteinases, e.g., matrix metalloproteinases (MMPs), to degrade provisional matrix [40, 41]; while depositing collagen and other extracellular matrix (ECM) components, e.g., proteoglycans, hyaluronic acid, glycosaminoglycans, and fibronectin, to form granulation tissue [5], which fill up the wound gap and provide a scaffold for cell adhesion, migration, growth and differentiation during wound repair [42, 43].

### Remodelling phase

The remodelling phase starts at the end of the granulation tissue development. Mechanical tension and cytokines, e.g., TGF-β, drive fibroblasts to differentiate into myofibroblasts, which express α-smooth muscle actin (SMA) and contract the wound [44]. Myofibroblasts undergo apoptosis when healing is complete [5]. At this phase, the quickly produced collagen III in the ECM is replaced by the collagen I, which has a higher tensile strength but takes longer time to deposit. The number of new blood vessels and the blood flow decline. A mature avascular and acellular environment is formed [45]. Some skin components, e.g., hair follicles and sweat glands, cannot be recovered after serious injury; and the healed skin can only achieve maximum ∼80 % of the original tensile strength [46].

## Factors regulating the transition from inflammation to proliferation during wound healing

Excessive and prolonged inflammation results in delayed healing and increased scar formation. However, compared with the knowledge about the initiation and amplification of inflammatory response, we know little about how inflammation is controlled and timely resolved, which is critical to enable progression into the proliferative phase. Here, we focus on reviewing factors deemed important for resolving inflammation and promoting proliferation, thus facilitating the transition from inflammation to proliferation during skin wound healing.

### Macrophages

In the intact skin, macrophages are the most abundant haematopoietic population, performing immune sentinel and homeostatic functions [47]. Upon skin injury, a large amount of monocytes exit the circulation and enter the wound site. Both infiltrating and skin-resident macrophages are activated by local microenvironmental signals and further develop into various subpopulations, defined by their distinct functional phenotypes [48]. PAMPs expressed by microbes and DAMPs produced during cellular stress in synergy with natural killer cell-derived IFN-γ polarize macrophages into classically activated macrophages (M1 subset), which promote Th1–Th17 immunity, host defense and antitumor immunity [49]. In contrast, cytokines such as IL-4 and IL-13 drive the formation of alternatively activated macrophages (M2 subset), which suppress inflammation and antitumor immunity, regulate glucose metabolism as well as facilitate wound repair [50–53]. TLR ligands together with immunoglobulin G immune complexes induce the development of regulatory macrophages, which produce IL-10 and TGF-β1 and play an immunosuppressive role [49]. This is to name just a few subpopulations with distinct functions and generally macrophages are considered to have the ability of either induce or suppress the immune response. Importantly, they manifest substantial plasticity in their phenotypes and functions, that is, they can readily switch from one functional phenotype to another in response to different microenvironment stimuli [54–56]. These properties make macrophages versatile players to assist and orchestrate all stages of wound healing (Fig. 2).

In the early phase of wound repair, upon exposure to pro-inflammatory cytokines, IFNs, microbial products or DAMPs, infiltrating monocytes and resident macrophages are activated and mainly acquire a pro-inflammatory M1 phenotype (Fig. 2) [57]. They perform phagocytosis of microbes, scavenging of dead cells and cellular debris and produce pro-inflammatory mediators, such as IL-1, IL-6, IL-12, TNFα, inducible nitric oxide synthase (iNOS), as well as chemokines to recruit additional leukocytes [10, 48, 58].

Later during the healing process, macrophages transit from a pro-inflammatory M1 to a reparative M2 phenotype, expressing anti-inflammatory mediators, e.g., IL-1R antagonist, decoy IL-1 receptor type II and IL-10, and growth factors, e.g., TGFβ, VEGF and IGF1, promoting fibroblast proliferation, ECM synthesis and angiogenesis (Fig. 2) [59, 60]. This M1–M2 transition is critical for the resolution of inflammation and tipping the balance to tissue repair [49]. As mentioned above, IL-4 and IL-13 are the canonical factors inducing the M2 phenotype, however, recent studies show that they are not necessary for modulating macrophage phenotypes in wounds in vivo [61, 62]. Anti-inflammatory cytokines (e.g., IL-10), glucocorticoids, prostaglandins, and modulators of glucose and lipid metabolism may also induce M2-like phenotypes. Importantly, the defection of M1–M2 phenotype transition of macrophages has been implicated in the pathogenesis of chronic wounds. For example, iron overload drives macrophages into an unrestrained pro-inflammatory M1-activation state, and iron-overloaded macrophages have been detected in human chronic venous ulcers [63, 64]. In a murine wound model iron overloaded macrophages were shown to impair skin wound healing [63]. In diabetes, up-regulation of proliferator-activated receptor (PPAR) γ activity, which is crucial for the switch in macrophage phenotype, was impaired by sustained expression of IL-1β in both mouse and human wounds. This results in deficient M1–M2 transition and is associated with delayed wound repair [65].

In the early inflammatory phase, neutrophils are abundant in the wound and they are essential for decontamination. However, if remaining in the wound over time, neutrophils cause more harm than good. For example, neutrophil proteases can degrade ECM as well as the proteins important for repair, e.g., clotting factors, complement, cytokines and immunoglobulin [66, 67]; they produce free oxygen radicals resulting in oxidative stress, which further damage tissue and delay healing [67]. Therefore, removal of neutrophils is prerequisite for wounds to progress into the proliferative phase. According to current knowledge, macrophages are the major players responsible for removing neutrophils in the wound: they induce neutrophil apoptosis [68]; then remove apoptotic neutrophils by phagocytosis, a process termed efferocytosis (Fig. 2) [69–72]. Interestingly, phagocytosis of neutrophils is also a central element to induce the phenotype switch of macrophages from pro-inflammatory M1 to a reparative M2 phenotype [73]. The importance of efficient removal of neutrophils by macrophages has been demonstrated in the cases of chronic wounds. Impairment of efferocytosis of macrophages, observed in diabetic wounds, prolongs the inflammatory phase and delays healing [70]. Moreover, in wounds of aged mice, the phagocytic function of macrophages was found deficient and associated with reduced healing [74].

In the final phase of healing, macrophages also regulate ECM content and remodeling by secretion of proteases and their inhibitors, such as matrix metalloproteinases (MMPs) and tissue inhibitors of metalloproteinase (TIMPs) [75–77]. Interestingly, a recent study revealed the important role of MMP-10 in regulating the collagenolytic activity of M2 macrophages, which is critical for scar resolution [41, 78]. A detailed discussion about the role of macrophages in fibrolysis was provided elsewhere [79].

Actually, the crucial role of macrophages in skin wound healing was established already in 1970s, when Leibovich and Ross showed that depletion of macrophages significantly delayed healing [80]. This finding has been confirmed later by use of genetically modified mice, allowing a highly specific depletion of macrophages in wounds [81, 82]. Recently, Lucas et al. temporally and selectively depleted macrophages at different phases of wound healing. Their study shows that when macrophages are absent in the inflammatory phase, re-epithelialization and granulation tissue formation are reduced; whereas depletion of macrophages in the proliferative phase results in haemorrhage [83]. In both conditions, progression to the following phases of wound repair fails [83].

Due to their crucial and pleiotropic roles during wound healing, macrophages become attractive therapeutic targets. Many attempts to improve healing have used in situ activation of macrophages in wounds (e.g., by topical treatment of wound with macrophage activating agent glucan [84]); recruitment of more macrophages (e.g., by application of monocyte chemoattractant protein-1 (MCP-1) to wounds [85]); or addition of exogenous macrophages [86, 87]. Although these strategies have shown to positively impact wound healing, increased amount of macrophages in wounds may imbalance the microenvironment and macrophages may switch to unfavorable phenotypes, e.g., pro-inflammatory instead of reparative phenotype [79]. Therefore, driving macrophages toward a reparative phenotype in wounds seems a particularly promising therapeutic approach.

### Stromal microenvironment

Stromal cells, such as fibroblasts, not only repair wounds by functioning as structure cells, they also actively regulate the inflammatory events [88, 89]. Fibroblasts can condition the local microenvironment and regulate the level and kinetics of inflammation, to match the severity of the damage. They interact with infiltrating inflammatory cells through CD40 receptors, which further activate the NF-κB signalling, causing fibroblasts to produce IL-6, IL-8, cyclooxygenase-2 and hyaluronan, regulating the infiltration and behavior of immune cells [90]. Cytokine deprivation induces apoptosis of inflammatory cells, which often happen at the peak of the inflammatory response, triggering resolution of inflammation [91]. Fibroblasts can regulate apoptosis of infiltrating immune cells by producing potent survival factors, e.g., type I IFNs [92]. Moreover, increased expression of stromal derived factor (SDF-1) from stromal cells and CXCR4 on infiltrating T cells have been observed in inflamed skin. The interaction between SDF-1 and CXCR4 may contribute to the inappropriate retention of immune cells in the skin [93]. Together, stromal cells play key roles in the inflammatory–proliferative phase transition: in addition to their ‘repair’ role, stromal cells also contribute to resolution of inflammation by withdrawal of survival factors and change of the chemokine gradient, leading to apoptosis or exit of the infiltrated immune cells from the skin.

### Bioactive lipid mediators

Fatty acids are not only part of the structural lipids in the skin; they are also the sources of a variety of bioactive lipids, e.g., eicosanoids, endocannabinoids and sphingolipids, regulating the inflammatory response and proliferation during wound healing [94].

Eicosanoids participate in initiation as well as resolution of inflammation. Upon injury, prostaglandins and leukotrienes are produced by infiltrating immune cells, which activate and amplify inflammation [95]. Prostaglandin E_2_ (PGE_2_) and D_2_ rapidly initiate the resolution process by inducing the expression of lipid mediators with both anti-inflammatory and pre-resolution functions, e.g., lipoxins [96, 97], resolvins and protectins [98–100], a process known as lipid-mediator class switch [97]. These lipid mediators can selectively stop neutrophil infiltration; increase monocyte recruitment and macrophage phagocytosis; stimulate the expression of genes important for antimicrobial defense; and promote the exit of phagocytes from the inflamed sites [100–103]. In addition to regulation of the inflammatory response, PGE_2_ has been shown to increase keratinocyte proliferation and migration, thus facilitating the transition to the proliferative wound healing phase [104]. In humans, eicosapentaenoic acid (EPA) and docosahexaenoic acid (DHA) supplementation increase eicosanoids, thus promoting wound re-epithelialisation [105]. Moreover, EPA and DHA have been shown to dampen the inflammatory response by competing with arachidonic acid in the lipoxygenase reaction, which leads to reduced production of pro-inflammatory lipid mediators [106].

Endocannabinoids, e.g., anandamide (AEA) and 2-arachidonoylglycerol (2-AG), bind to their G-protein-coupled cannabinoid (CB) receptors and play anti-inflammatory roles in the skin [94]. For example, AEA suppresses keratinocyte production of TNF-α and MCP-1 [107]. In addition it inhibits T cell proliferation and production of TNF-α and IFN-γ by CD4+ and CD8+ T cells and IL-17 by Th17 cells [108]. AEA has also been shown to suppress mast cell numbers and activity in human skin [109]. 2-AG increases the number of phagocytosing macrophages, which leads to increased production of anti-inflammatory cytokines, e.g., TGF-β1 and decreased output of pro-inflammatory cytokines, e.g., TNF-α by macrophages [73]. Moreover, the reactive oxygen species (ROS) production by macrophages is also regulated by the balance of CB1 and CB2 activation, which is an important factor contributing to the persistent inflammation in chronic wounds and increasing the senescence of dermal fibroblasts [63, 110]. The specific role of endocannabinoids in skin wound healing remains largely unexplored [94]. A relevant research regarding periodontal healing has demonstrated increased expression of CB1 and CB2 on fibroblasts and macrophages in granulation tissue, as well as higher levels of AEA in gingival crevicular fluid after wounding [111]. The activation of endocannabinoid signalling is important for proliferation of gingival fibroblasts [111].

Sphingolipids play a broad role in the skin and some sphingolipid metabolites have been postulated as potential therapeutic targets for chronic wounds [94]. For example, sphingosine-1-phosphate, produced by platelets at the haemostasis phase of wound healing, has been shown to promote keratinocyte migration and wound healing [112–114]. Sphingosylphosphorylcholine increases proliferation of human keratinocytes, and induces the production of wound healing factors by human fibroblasts, e.g., connective growth tissue factor, IL-6 and plasminogen activator inhibitor-1 [115–118].

Together, in addition to the protein mediators, i.e., cytokines and chemokines, bioactive lipid mediators are important players regulating the transition from the inflammatory to the proliferative phase of wound healing.

### Redox signals

During normal metabolic processes reactive oxygen species (ROS) are produced by all cells. In wounds, increased amounts of ROS (e.g., superoxide anion, hydroxyl radicals, singlet oxygen, hydrogen peroxide) are produced by NADPH oxidase, an enzyme complex highly expressed in inflammatory cells [119]. ROS can damage cells by oxidation of cellular macromolecules, thus normally they are rapidly detoxified by catalase, peroxidases, peroxiredoxins and low molecular weight antioxidants [119]. The classical view about the role of ROS in wound healing is to protect the host against invading bacteria and other microorganisms. However, recent studies reveal that low levels of ROS can also function as mediators of intracellular signalling, playing crucial roles throughout the healing process (reviewed in [120]). In the hemostasis phase, ROS regulate blood coagulation, thrombosis and platelet functions. In the inflammation phase, in addition to being antimicrobial, ROS enhance the recruitment and function of leukocytes. In the proliferation phase, low concentrations of ROS have been shown to induce proliferation and migration of epithelial cells. Moreover, Roy et al. found that low concentrations of H_2_O_2_ supported healing by promoting angiogenesis, whereas higher doses of H_2_O_2_ adversely influenced healing [121]. Tight control of redox signals is crucial for the transition from inflammation to proliferation during wound healing. Excessive amounts of ROS cause oxidative stress, which damage cells and are observed in chronic hard-to-heal wounds [119].

### Negative regulation of TLR signalling

In skin wound healing, TLRs are the most well characterized receptors on host cells, recognizing danger signals, i.e., invading pathogens and tissue debris, and initiating inflammatory response to remove these danger signals. However, TLR-induced inflammation needs to be resolved after removal of the danger signals, to allow wound healing to proceed. The process of inflammation resolution involves not only passive mechanisms, e.g., dissipation of chemotactic gradient or initial danger signals, but also active biochemical pathways [103]. In the case of TLR signalling, a plethora of inhibitory mechanisms have been discovered. Interestingly, most of these inhibitors are induced through TLR activation, thus acting through a negative-feedback loop to limit or turn off the TLR signalling [122]. The molecular mechanisms inhibiting TLR signals (Fig. 1) include (a) interference of ligand binding, e.g., soluble forms of TLR2 and TLR4 have been identified to function as decoy, competing with the membrane-bound forms of TLRs for ligands binding [123, 124]; (b) reduction of TLR expression, e.g., anti-inflammatory cytokine TGF-β suppresses the expression and function of TLR4 [125]; (c) degradation of TLRs, e.g., Triad3A can bind to the cytoplasmic domain of TLR4 and TLR9 and promote their ubiquitylation and degradation [126]; (d) inhibition of TLR downstream signalling, e.g., suppressor of cytokine signalling 1 (SOCS1), interleukin-1 receptor-associated kinase M (IRAKM), Toll-interacting protein (TOLLIP), IRAK2c and IRAK2d have been shown to specifically suppress the function of IRAK family of kinases; a cysteine protease enzyme A20 has been shown to block TLR-mediated signalling by deubiquitylating TNF receptor-associated factor (TRAF) 6; both IRAK and TRAF6 are the key players in the TLR signalling pathways [122]; (e) change of structures of target genes through chromatin remodelling and histone modification, e.g., H2AK119 ubiquitylation and H3K27 trimethylation inhibit the expression of TLR-signal-targeted genes [127]. Recently, TLR signalling has also been shown to be regulated by microRNAs at multiple levels, such as by targeting TLRs, downstream signalling proteins, related regulatory molecules, transcription factors as well as genes induced by TLR signalling (e.g., cytokines), which was reviewed elsewhere [128]. Deletion of genes encoding these inhibitors results in a hyperinflamed state. For example, in mice with deficient dual specificity phosphatase 1 (DUSP1) expression, a MAPK phosphatase regulating TLR signalling, lipopolysaccharide (LPS) challenge induces overshooting production of IL-6 and TNF-α and increased infiltration of neutrophils [129].

Although mounting evidence has shown important roles for TLR signalling in physiological wound healing, their expression and function in chronic wounds remain largely unknown [130]. In diabetic mouse, deletion of TLR2 decreased inflammation and accelerated wound closure, suggesting that excessive TLR2 signalling may be detrimental to diabetic wounds [131]. In line with this finding, Pukstad et al. reported that human non-healing venous ulcers were associated with persistent activation of TLR2 and TLR4 signals [132]. It is unknown whether the excessive TLR signalling in chronic wounds is due to the impairment of inhibitory mechanisms as aforementioned, which warrant future investigation.

### Transcription factors

Transcription factors orchestrate the dynamic and complex gene expression programs during wound healing. Here we focus on the transcription mechanisms functioning in both the inflammatory and proliferative phases of skin wound healing, since changes of these mechanisms may affect phase transition (Table 1). Extensive review regarding the function of transcriptional factors in wound repair in general can be found elsewhere [133–135].

**Table 1:** Transcription factors regulating inflammation and proliferation in skin wound healing

Transcription factor	Inflammation	Proliferation			References
		Re-epithelialization	Granulation tissue	Angiogenesis	
GRs	**−**	**−**	**−**		[138, 139]
ARs	**+**	**−**			[140–143]
ERs	**−**	**+**			[144–147]
PPARs	**−**	**+**			[149–151]
AP-1	**−**	**+**			[152–155]
E2F1	**+**	**+**			[157]
Smad2		**−**			[159]
Smad3	**+**	**−/+**			[160–162]
Smad4	**−**	**+/−**			[163, 164]
Smad7	**−**	**+**		**−**	[165]
EGR1	**+**	**+**	**+**	**+**	[166–168]
HoxD3		**+**	**+**	**+**	[171]
HoxA3		**+**	**+**	**+**	[172]
HoxB13	**+**	**−**		**+**	[173, 174]

*+* positive regulation, *−* negative regulation

#### Glucocorticoid receptors

As shown in several experimental and clinical studies, glucocorticoids inhibit wound healing, which is due to their anti-inflammatory and anti-mitotic effects on several cell types in the wounds [136]. Glucocorticoids bind to and activate glucocorticoid receptors (GRs), which migrate to the cell nucleus, form homodimers and bind to specific DNA-binding elements, i.e., glucocorticoid response elements, in the promoter or enhancer regions of target genes [137]. In addition, glucocorticoids regulate gene transcription through interacting ligand-receptor monomers with members of the activating protein 1 (AP-1) or NF-κB transcription factor families [137]. To characterize the endogenous role of glucocorticoid in wound healing, the mouse with GRs lacking DNA-binding capacity was generated. In the wounds of these mice, there are increased number of inflammatory cells and high level of IL-1β. Also, formation of granulation tissues in these mice is accelerated, with enhanced proliferation and migration of fibroblasts, which is in line with the anti-fibrogenic activity of glucocorticoids [138]. On the contrary, keratinocyte-targeted overexpression of GRs leads to delayed re-epithelialization and granulation tissue formation, which is accompanied by reduced expression of pro-inflammatory cytokines and infiltration of granulocytes and macrophages in the wounds [139].

#### Androgen and estrogen receptors

It has been observed that there are sex differences in wound repair and males have a higher risk to develop chronic wounds than females, which may be due to different effects of androgens and estrogens on wound healing [140, 141]. Both androgens and estrogens bind to nuclear hormone receptors, i.e., androgen receptors (ARs) and estrogen receptors (ERs), which further interact with specific DNA-binding elements, regulating target gene expression [134]. Castration of male mice or inhibition of the conversion of testosterone to 5α-dihydrotestosterone (DHT) reduce the production of pro-inflammatory cytokines and accelerate re-epithelialisation of skin wounds, whereas treatment with DHT decreases the migratory capacity of keratinocytes [140, 142]. In line with this, treatment of wounds with androgen receptor antagonist promotes healing, supporting the negative effects of androgens on skin wound healing [143]. In contrast to androgens, estrogen has been demonstrated as a positive effector in wound healing. It has been found that the wound heals slower in aged women and also in ovariectomized young female rodents [144]. Topical application of estrogens or ER agonists in both aged females and males can improve healing, which may be partially due to the reduced expression of macrophage migration inhibitory factor (MIF) and activation of keratinocytes [145–147]. Together, the balance between estrogen and androgen signalling may impact the outcome of wound healing.

#### Peroxisome proliferator-activated receptors

The peroxisome proliferator-activated receptor (PPAR) family, consists of three members PPARα PPARβ/δ and PPARγ, which are activated by polyunstaturated fatty acids and different fatty acid derivatives [148]. Delayed wound healing has been found in PPARα −/− and PPARβ/δ +/− mice, but not in PPARγ +/− mice [149]. Loss of PPARα in mouse leads to exaggerated inflammation in wounds, indicating its anti-inflammatory function [149]. Upon wounding, PPARβ/δ has been shown to promote re-epithelialisation by increasing migration while decreasing apoptosis of keratinocytes [149, 150]. PPARβ/δ also exerts an anti-inflammatory effect by inducing the expression of IL-1 receptor antagonist in fibroblasts [151].

#### Activator protein 1

Activator protein 1 (AP-1) leucine zipper transcription factors, including hetero- or homodimers of the Fos, Jun and CREB/ATF protein families, have been shown to be up-regulated and/or activated after injury, which is important for re-epithelialisation [152, 153]. Mice heterozygous for the nuclear hormone receptor coactivator, which activates c-Fos and c-Jun, develop chronic wounds with reduced migration of keratinocytes [154]. Upon injury, mice with deficient expression of JunB in the skin show prolonged inflammation, epidermal hyperproliferation and aberrant differentiation [155].

#### E2F transcription factors

E2F transcription factor family is key regulators of cell proliferation and embryogenesis [156]. The expression of E2F1 and E2F2 has been found to be up-regulated in wound-edge keratinocytes in healing human venous stasis ulcers [157]. Accordingly, lack of E2F1 in mouse skin results in delayed inflammatory response and re-epithelialisation [157].

#### Smad proteins

TGF-β are pleiotropic cytokines regulating all phases of wound healing [158]. TGF-β binds to the constitutively active TGF-β type II receptor, which dimerizes, phosphorylates and activates type I receptor. Receptor dimerization further activates Smads 2 and 3, which interact with Smad4 and enter the nucleus. The Smad complex binds to the Smad-binding element on the target genes and regulates their transcription. This signalling pathway is subjected to sophisticated regulation, including the inhibitory Smads, i.e., Smad6 and Smad7 [158]. It has been shown that transgenic mice overexpressing Smad2 in basal keratinocytes suffer from delayed wound healing, due to reduced keratinocyte migration [159]. The role of Smad3 in wound healing remains controversial: accelerated re-epithelialisation has been observed in Smad3 knockout mice, which is accompanied with reduced inflammatory response [160, 161]; however, Sumiyoshi et al. showed that subcutaneous injection of Smad-3-expressing adenovirus enhanced re-epithelialization in a rabbit ulcer model [162]. In this study, the adenovirus mainly targeted fibroblasts, whereas in Smad3 knockout mice all cells were affected, which may account for the discrepancy between these results. Smad4 knock-out mice have been established in two independent studies: Owens et al. used a mouse model with deficient Smad4 in multiple tissues/cell types, e.g., skin, erythrocytes, and B and T cells, and found that lack of Smad4 resulted in delayed wound closure accompanied with increased inflammation [163]; whereas Yang et al. used a mouse model with specific deletion of Smad4 in epidermal keratinocytes and showed enhanced re-epithelialisation of wounds, which was mainly due to increased keratinocyte proliferation [164]. The discrepancy of the results indicates a complex role of Smad signalling depending on the cellular context. The expression of inhibitory Smad, Smad7, was shown to be increased during wound healing [165]. Transient overexpression of Smad7 in epidermal keratinocytes results in accelerated re-epithelialization due to increased keratinocyte proliferation and migration; as well as reduced inflammation and angiogenesis through indirect effects on the wound stroma [165].

#### Early growth response 1

Early growth response 1 (EGR1) is a zinc finger transcription factor essential for skin wound healing in adults. Its deletion leads to impaired migration of keratinocytes and inflammatory cells, reduced myofibroblast differentiation and thus delayed healing [166, 167]. In contrast, overexpression of EGR1 in wounds of adult mice and rats increased re-epithelialisation, angiogenesis, collagen deposition and wound contraction by up-regulating several growth factors [168]. Moreover, transgenic mice overexpressing EGR1 in fibroblasts exhibited exuberant tissue repair with increased collagen deposition and tensile strength of the wounds [166]. Interestingly, although the expression of EGR1 was increased in the wound edge of E11.5 mouse embryos, its deletion did not affect embryonic wound healing [169]. Wound healing without scarring (i.e., scarless healing) is observed only in embryos, and is lost after birth. EGR1 may be mechanistically involved, contributing to the difference between embryos and adults in this respect.

#### Homeobox genes

Homeobox genes are a family of highly conserved transcription factors regulating organ patterning during development. Recently, several homeobox genes were shown important in skin wound healing [170], such as HoxA3 and HoxD3 improving healing in diabetic mice by enhancing re-epithelialization, angiogenesis and collagen deposition [171, 172]. However, HoxB13 negatively affects wound healing: its overexpression delayed wound healing, accompanied with prolonged inflammation [173]; whereas knockout of HoxB13 facilitates healing [174]. It is noteworthy that HoxB13 is down-regulated in embryonic scarless wound healing, but not in adult wounds [174], indicating that differential expression of transcriptional factors may account for the difference in healing between embryo and adult.

### Epigenetic regulatory mechanisms facilitating the inflammation–proliferation transition

Epigenetics entails studying heritable changes of gene expression, which result in alterations of the phenotype without affecting the genomic DNA sequence [175]. Epigenetic mechanisms control gene expression at different levels, i.e., covalent DNA and histone modifications (e.g., DNA methylation, histone methylation, phosphorylation and acetylation), ATP-dependent and higher-order chromatin remodelling, as well as non-coding RNA and microRNA-mediated regulation [176]. Different epigenetic regulators exhibit dynamic expression patterns during wound healing. For example, polycomb group (PcG) proteins form polycomb repressive complexes (PRCs), which are recruited to chromatin, modify histone and suppress gene expression [177, 178]. During wound healing, the expression of PcG proteins, e.g., enhancer of zeste 2 polycomb repressive complex 2 Subunit (EZH2), SUZ12 and EED are transiently downregulated, whereas histone demethylases lysine (K)-specific demethylase 6B (KDM6B) and 6A (KDM6A) are up-regulated [179]. This indicates that the loss of PcG proteins-mediated suppression may transiently activate a group of genes, participating in skin repair. However, the exact role of epigenetic regulation in wound healing remains largely unexplored.

Epigenetic modifications regulate the expression of genes important for resolution of inflammation. It has recently been shown that in dendritic cells and macrophages treated with LPS, loss of TET2, an enzyme that catalyzes DNA demethylation, results in prolonged and high expression of proinflammatory cytokines, including IL-6 [180]. In the diabetic mouse model, there is enhanced recruitment of histone methyltransferase SET7 to the MCP-1 promoter region, which results in increased histone H3-lysine 4 methylation and higher MCP-1 expression in macrophages treated with TNF-α [181]. In diabetic wounds, histone methylation, i.e., a repressive histone methylation marker, H3K27me3, was found to be decreased at the promoter of the IL-12 gene, which may drive macrophages toward an inflammatory phenotype [182].

Epigenetic modifications also play important roles in regulating the proliferative phase of wound healing. It has been found that deletion of PcG proteins EZH1 and EZH2 resulted in defective proliferation of stem cells and thus impaired wound healing [183]. Also, administration of DNA methyltransferases (DNMT) inhibitor, 5-aza2′-deoxycytidine, and histone deacetylase (HDAC) inhibitor, Trichostatin A (TSA) to mice that had digit amputation promotes wound healing by increasing proliferation of stem cells [184]. Although studies directly examining the role of epigenetic modifications in keratinocyte migration are lacking, several research groups have reported that the expression of MMPs, which are the enzymes degrading ECM and allowing cell migration, are regulated by epigenetic mechanisms [185–188]. Moreover, HDAC has been shown to regulate both collagen production by fibroblasts and TGF-β1-mediated myofibroblast transformation [189–192]. An altered pattern of DNA methylation and histone acetylation has been identified in keloid fibroblasts compared with fibroblasts from normal scars [193]. Inhibition of HDAC with TSA leads to decreased collagen synthesis and increased apoptosis of keloid fibroblasts, indicating that epigenetic modification may be a potential treatment for keloid [194]. Epigenetic mechanisms also regulate angiogenesis. For example, inhibition of HDAC attenuates VEGF signalling, which is important for vascular morphogenesis and endothelial differentiation [195]. PcG proteins, e.g., EZH2, are up-regulated and involved in VEGF-mediated stimulation of angiogenesis [196, 197].

Together, epigenetic regulators play important roles in skin wound healing, especially in the inflammatory–proliferative phase transition. Future research in this area may identify epigenetic modulating drugs, which may be used to treat impaired wound healing.

### MicroRNAs

In the human genome, the majority of the transcriptional output is constituted by RNAs that lack protein-coding capacity [198, 199]. Intensive research in the recent decade has revealed that these non-coding RNAs (ncRNAs) function as important regulators of cellular physiology and pathology, which makes them promising therapeutic and diagnostic entities. MicroRNAs (miRNAs) are ~22 nt single-stranded RNAs and are the most well-known ncRNAs to date. They incorporate into the RNA-induced silencing complex (RISC) and bind to the 3′ untranslated region (UTR) of the target mRNA, which results in translational repression or degradation of target mRNAs [200]. MiRNA are proposed to regulate the majority of protein-coding genes in humans [201]. Deregulation of miRNA expression has been shown to contribute many diseases.

MiRNAs are key regulators controlling mammalian skin development and morphogenesis, which has been demonstrated by conditional knock-out of enzymes essential for miRNA biogenesis, e.g., Dicer, Drosha or DGCR8 [202–204]. To date, miRNAs have been shown to play important roles in a variety of physiological and pathological processes in the skin (reviewed in [205]), many of which constitute key steps of wound repair, e.g., proliferation, migration and angiogenesis. Recently, Ghatak et al. revealed increased Dicer expression and bolstered miRNA biogenesis at the later phase of healing process [206]. Keratinocyte-specific depletion of Dicer in mice prior to wounding compromised wound closure, indicating a critical role for miRNA-mediated regulation of wound healing [206]. Here we primarily focused on the miRNAs that have been demonstrated in vivo to directly regulate the inflammatory and proliferative phases during skin wound healing (Table 2).

**Table 2:** MicroRNAs regulating inflammation and proliferation in skin wound healing

MiRNA		Expression in WH^a^	Cell type	Function	Target	References
Inflammatory phase						
mir-146a		Down^b^	Macrophage, keratinocyte	A brake to prevent excessive inflammatory response and contribute to resolution of inflammation	IRAK1, IRAK2, TRAF6	[207–212]
mir-132		Up^b^	Keratinocyte	Decreases keratinocytes chemokine production and capability of to attract leukocytes	HB-EGF	[209]
mir-155		Up^b^	Macrophage	Inhibition of mir-155 improves healing by reducing inflammation and fibrosis.	BCL6^c^, RHOA^c^, SHIP1	[209, 217, 218]
mir-21		Up^b^	Macrophage	Turns on anti-inflammatory phenotype in the postefferocytotic macrophage	PTEN, GSK3β, PDCD4	[220–222]
Proliferative phase						
mir-132		Up^b^	Keratinocyte	Promotes keratinocyte growth	HB-EGF	[209]
mir-21		Up	Keratinocyte, fibroblast	Regulates cell migration, re-epithelialization, wound contraction and collagen deposition	TIMP3, TIAM1	[221–223, 240]
mir-130a		Increased in chronic wounds	Keratinocyte	Delays re-epithelialization	LepR^c^	[223]
mir-31		Up^b^	Keratinocyte	Promotes keratinocyte proliferation and migration	EMP1	[224]
mir-483-3p		Peaks at final stage of WH	Keratinocyte	Inhibits keratinocyte migration and proliferation	MK2, MKI67, YAP1	[225]
mir-203		Down	Keratinocyte	Suppresses keratinocyte proliferation and migration	RAN, RAPH1, TP63, LASP1	[229]
mir-99 family		Down^b^	Keratinocyte	Promotes keratinocyte proliferation and migration	IGF1R, mTOR, AKT1	[209, 230]
mir-198		Down	Keratinocyte	Suppresses keratinocyte migration	DIAPH1, PLAU, LAMC2	[231]
mir-210		Down^b^, increased in ischemic chronic wounds	Keratinocyte	Inhibits keratinocyte proliferation	E2F3	[232]
			Endothelial cells	Promotes angiogenesis	EFNA3	[233]
mir-146a		Induced by biofilm-infection	Keratinocyte	Impairs tight junction function	ZO-1, ZO-2	[234]
mir-106b						
mir-27b		Down^b^	Vascular precursor cells	Promotes angiogenesis in diabetic wounds	TSP-1, SEMA6Ap66Shc	[209, 236]
			Mesenchymal stem cell (MSC)	Inhibits mobilization of MSCs to wounds and delays healing	SDF-1α	[237]
mir-200b		Down	Endothelial cells	Suppresses angiogenesis	GATA2, VEGFR2	[238]
mir-378a		Unknown	Fibroblast	Inhibition of mir-378a promotes fibroblast migration and wound closure.	VIM, ITGB3	[239]
mir-196a		Unknown	Fibroblast	Suppresses collagen production	COL1A1, COL3A1	[241]

^a^WH, wound healing

^b^Up, up-regulated; down, down-regulated at the inflammatory phase of normal wounds compared with the intact skin

^c^BCL6, B-cell CLL/Lymphoma 6; RhoA, Ras homolog family member A; LepR, leptin receptor

#### MiRNAs regulate inflammation during wound healing

MiR-146a, miR-132 and miR-155 are the first miRNAs identified to be associated with inflammation, induced in a monocytic cell line treated with TLR4 ligand, LPS [207]. Recently, Meisgen et al. showed that miR-146a expression was also increased in keratinocytes treated with TLR2, TLR3 or TLR5 ligands [208]. During human skin wound healing, miR-146a was down-regulated in the inflammatory phase [209]. MiR-146a was found to negatively regulate the innate immune response by targeting IRAK1, IRAK2 and TRAF6 in monocytes, macrophages and epidermal keratinocytes, suggesting that it may act as a brake to prevent excessive inflammatory response and contribute to resolution of inflammation [207, 210, 211]. In line with this, decreased miR-146a and enhanced pro-inflammatory target genes were observed in skin wounds of diabetic mouse model [212].

MiR-132 is one of the top up-regulated miRNAs in the inflammatory phase of human skin wounds in vivo, which level further peaks in the subsequent proliferative phase [209]. It has been shown that miR-132 decreases the production of chemokines and the capability of keratinocytes to attract leukocytes by suppressing the NF-κB signalling pathway. Heparin-binding EGF-like growth factor (HB-EGF) was identified as a key target mediating the biological functions of miR-132 in keratinocytes [209].

MiR-155 is a critical regulator in the development of immune cells [213]. The expression of miR-155 was highly induced in macrophages stimulated with inflammatory cytokines or TLR ligands [214]. Overexpression of miR-155 decreases the expression of its target Src homology-2 domain-containing inositol 5-phosphatase 1 (SHIP1) in hematopoietic cells, which leads to increased activation of the kinase Akt during the cellular response to LPS [215]. In line with this, Tili et al. reported that miR-155 enhanced TNFα translation by targeting several LPS signalling mediators in macrophages, e.g., Fas-associated death domain protein (FADD), licB kinase epsilon (IKBKE), and receptor (TNFR superfamily) interacting serine-threonine kinase I (RIPK1) [216]. During skin wound healing, miR-155 was found to be upregulated in wounds in the inflammatory phase in both human and mouse [209, 217]. MiR-155 knock-out mice revealed enhanced wound closure compared to wild-type mice [217]. In line with this, Yang et al. injected miR-155 antagomir into the wound edge of mouse and found that antagonizing miR-155 locally improved healing by reducing fibrosis and inflammation [218].

MiR-21 has been proposed to function in dampening inflammation [219]. MiR-21 is induced in macrophages by resolving D1, which is an endogenous lipid mediator generated during the resolution phase of acute inflammation. In addition, Das et al. revealed that miR-21 plays an important role in regulating engulfment of apoptotic cells by macrophages, which is prerequisite for the timely resolution of inflammation during wound healing [220]. The expression of miR-21 is induced in macrophages by efferocytosis, which tempers the LPS-induced inflammatory response by silencing its targets phosphatase and tensin homolog (PTEN) and glycogen synthase kinase (GSK) 3β. Moreover, miR-21 silences programmed cell death protein (PDCD) 4, favoring c-Jun-AP-1 activity, which in turn results in elevated production of anti-inflammatory IL-10 by macrophages. Together, this work indicates that miRNAs are important in turning on an anti-inflammatory phenotype in the postefferocytotic macrophage [220].

#### MiRNAs regulate the proliferative phase during wound healing

##### Re-epithelialization

In addition to its role in regulating wound inflammation, miR-132 also promotes keratinocyte growth through targeting HB-EGF and increasing the activity of the signal transducer and activator of transcription (STAT) 3 and MAPK signalling pathways [209]. Using mouse in vivo and human ex vivo wound model, we showed that miR-132 blockade delayed healing, which was accompanied by severe inflammation and deficient keratinocyte proliferation. These results indicate that miR-132 is a critical regulator that may facilitate the transition from the inflammatory to the proliferative phase during wound healing [209].

MiR-21 is another player regulating both the inflammatory and proliferative phases of wound healing. MiR-21 was found up-regulated after wounding, mainly in activated and migrating epithelial cells of the epidermis and in dermal mesenchymal cells [221, 222]. Yang et al. revealed that miR-21 expression was induced by TGF-β1 in keratinocytes, promoting keratinocyte migration by targeting TIMP3 and T-cell lymphoma invasion and metastasis (TIAM) 1 [222]. Antagonizing miR-21 locally in the wound was shown to delay wound re-epithelialization [222] and impair wound contraction and collagen deposition [221]. Interestingly, Pastar et al. found that miR-21 was overexpressed in human chronic non-healing wounds compared to healthy skin. Local overexpression of miR-21 delayed re-epithelialization in a human skin ex vivo wound model, as well as inhibited re-epithelialisation and granulation tissue formation in a rat wound model, indicating the importance of controlling the expression of miR-21 in wounds [223]. In the same study, Pastar et al. also reported that miR-130a, which is increased in human chronic wounds, delayed re-epithelialisation [223].

In human skin wounds, miR-31 was shown to be gradually up-regulated in wound-edge keratinocytes in the inflammatory through the proliferative phase in comparison with intact skin [224]. MiR-31 promoted keratinocyte proliferation and migration by silencing epithelial membrane protein 1 (EMP-1), suggesting a positive role in regulating re-epithelialization [224].

In contrast, miR-483-3p, which level peaks at the final stage of the wound closure, has been shown to inhibit keratinocyte migration and proliferation by down-regulating its targets mitogen-activated protein kinase-activated protein kinase (MK) 2, marker of proliferation Ki-67 (MKI67), and yes-associated protein (YAP) 1, representing a novel mechanism for controlling keratinocyte growth arrest in the final steps of re-epithelialization [225].

MiR-203 is the most abundant keratinocyte-specific miRNA in the epidermis [226]. It controls stemness and is fundamental for skin development and differentiation [227, 228]. In wounded mouse epidermis, miR-203 was found down-regulated in the proliferating keratinocytes of the ‘migrating tongue’, whereas it is strongly expressed in the differentiating cells of the skin outside the wound. Accordingly, miR-203 was shown to suppress keratinocyte proliferation and migration by regulating its targets Ras-related nuclear protein (RAN), Ras association (RalGDS/AF-6) and pleckstrin homology domains (RAPH) 1, Tumor protein P63 (TP63) and LIM and SH3 protein (LASP) 1, indicating its possible role in wound re-epithelialization and epidermal homeostasis re-establishment of injured skin [229].

In both human and mouse wound models, miR-99 family (miR-99a, miR-99b and miR-100) were found downregulated in the inflammatory phase [209, 230]. Jin et al. showed that downregulation of the miR-99 family members leads to activation of AKT/mTOR signalling pathway by targeting insulin-like growth factor 1 receptor (IGF1R), mechanistic target of rapamycin (mTOR) and V-Akt Murine Thymoma Viral Oncogene Homolog (AKT) 1, which in turn activates keratinocyte proliferation and migration, and facilitates wound closure [230].

MiR-198 has been shown to suppress keratinocyte migration by targeting and inhibiting diaphanous-related formin (DIAPH) 1, plasminogen activator urokinase (PLAU) and laminin gamma (LAMC) 2 [231]. Interestingly, this miRNA is coded in the 3′-untranslated region of follistatin-like 1 (FSTL1) mRNA, which promotes cell migration. Upon wounding, the expression of FSTL1 is switched on, while the miR-198 expression is turned off, due to the TGFβ-mediated down-regulation of a KH-type splicing regulatory protein (KSRP), which is essential for processing the primary transcript into miR-198. Failure of this switch, as shown in chronic diabetic ulcers, in which expression of miR-198 persists while FSTL1 is absent, leads to impaired keratinocyte migration and re-epithelialization [231].

In ischemic chronic wounds, high level of hypoxia inducible factor-1α (HIF-1α) is present. HIF-1α has been shown to induce the expression of miR-210, a miRNA inhibiting keratinocyte proliferation by targeting cell-cycle regulatory protein E2F3 [232]. Moreover, hypoxia-induced miR-210 in endothelial cells has been shown to promote angiogenesis by targeting Ephrin-A3 (EFNA3) [233].

Infection is another complication in chronic wounds, where bacteria may form biofilms, which makes them recalcitrant to antimicrobials and host defense. Using a porcine burn wound model, Roy et al. showed that biofilm-infection induce miR-146a and miR-106b in the wound-edge tissue, which targets zona occludens (ZO)-1 and ZO-2 in keratinocytes to compromise tight junction function [234]. This is relevant to the clinical observation that even if an infected wound seems healed macroscopically it may still be compromised by impaired epidermal barrier function and risk for re-infection.

##### Angiogenesis

The roles of miRNAs in angiogenesis/neovascularization have been intensively investigated, and was reviewed elsewhere (such as in [235]). Here we focus only on the studies relevant for skin wound healing.

In diabetes mellitus, the angiogenic potential of vascular precursor cells is impaired. Wang et al. reported that vascular precursor cells from mouse model of type 2 diabetes have lower miR-27b expression compared to the normal littermate mice. Overexpression of miR-27b improves the functions of vascular precursor cells in diabetic mice, i.e., proliferation, adhesion, tube formation, and delays apoptosis by targeting thrombospondin-1 (TSP-1), semaphorin 6A (SEMA6A), and pro-oxidant protein p66Shc. Importantly, topical cell therapy with diabetic vascular precursor cells overexpressing miR-27b or direct local miR-27b delivery enhances diabetic skin wound closure, with a concomitant augmentation of wound perfusion and capillary formation [236]. However, in burn wounds, Lv et al. found that miR-27b expression was decreased compared to intact skin. MiR-27b targets stromal cell-derived factor-1α (SDF-1α), a chemokine crucial for the recruitment of mesenchymal stem cells (MSCs) from bone marrow reservoirs to repair wounds. Injection of miR-27b into the burn wound edge inhibited the mobilization of MSCs to the epidermis and delayed healing [237].

Moreover, Chan et al. found that wounding transiently down-regulated miR-200b in wound-edge endothelial cells, which lead to increased expression of its targets, e.g., globin transcription factor binding protein 2 (GATA2) and vascular endothelial growth factor receptor 2 (VEGFR2), and switch on angiogenesis. This process is impaired in diabetic wounds, in which excessive TNF-α increases miR-200b expression, silences GATA2 and VEGFR2, and suppresses angiogenesis [238].

##### Granulation tissue formation

MiR-378a has recently been shown to negatively affect skin wound healing: inhibition of miR-378a enhances wound healing in vivo, which leads to accelerated fibroblast migration and differentiation by targeting vimentin (VIM) and β3 integrin (ITGB3) [239]. As aforementioned, the increased expression of miR-21 during normal wound healing also promotes fibroblast migration [240]. Moreover, miR-196a has been shown to regulate collagen production by fibroblasts through directly targeting collagen type 1 alpha 1 (COL1A1) and COL3A1 [241].

Studies using animal models and recent clinical trials demonstrate that modulation of miRNA expression by administration of specific miRNA mimics/inhibitors may be used therapeutically [242]. Importantly, modulating miRNAs may be a more effective strategy than traditional drugs targeting single proteins, since a single miRNA can work as a switch, regulating an entire functional network. Therefore, modulation of miRNAs holds great promise to be effective treatments improving wound healing.

## Wound complications associated with deficient transition from inflammation to proliferation

Accumulating evidence associates excessive inflammation with major wound healing disorders, e.g., chronic wounds and hypertrophic or keloid scars. This provides a rationale for targeting the inflammatory–proliferative phase transition to improve the outcome of wound healing.

### Chronic wound

Chronic wounds present a major, and increasing, health and economic burden to our society [3]. It often occur in patients with one or several underlying disorders, e.g., venous or arterial insufficiency, diabetes mellitus or systemic inflammatory disease, all of which generate an unfavorable milieu for the delicate repair process, trapping wounds in a constant inflammatory state and failing to progress through the normal healing stages [2]. Persisting inflammatory cells, mainly neutrophils and macrophages, generate large amount of proinflammatory cytokines and a highly proteolytic microenvironment at the wound site [6]. Proinflammatory cytokines strongly induce the expression of various MMPs, and down-regulate the expression of TIMPs [243, 244]. In addition, the neutrophil-originated serine proteinases (SPs) are highly expressed in chronic wounds [245, 246]. As a consequence, factors crucial for wound healing, e.g., the major proteinase inhibitors (e.g., α1-proteinase inhibitor and α2-macroglobulin), components of the provisional wound matrix (e.g., fibronectin and vitronectin), growth factors (e.g., PDGF, VEGF) are degraded or inactivated by proteolytic cleavage in chronic wounds [246, 247]. Moreover, increased ROS in the chronic wound, mainly from neutrophils, damages cell membranes and structural proteins of the ECM, as well as promotes the production of proinflammatory cytokines (e.g., IL-1, IL-6, TNF-α), chemokines and proteolytic enzymes (e.g., MMPs and SPs), amplifying the unrestrained inflammation [248]. Non-healing wounds are often complicated with bacterial infection, which sustains a continuous influx of neutrophils and macrophages and further delays wound repair [249]. Chronic wounds are difficult to treat due to their complex nature and our limited understanding of pathogenetic mechanisms. Up to one-third of treated patients experience four or more episodes of recurrence [3]. Therefore, identification of new therapeutic targets is needed. In this respect modification of factors facilitating the transition from inflammation to proliferation is likely a fruitful strategy for re-setting the wound environment and activating tissue regeneration.

### Bacteria and wound healing

Bacteria exist in all open skin wounds and the interaction between bacteria and host ranges from contamination (i.e., the presence of non-replicating bacteria) through colonization (i.e., the presence of replicating bacteria, but absence of tissue damage) on to local infection (i.e., the presence of replicating bacteria with subsequent host injury), and finally to spreading infection which manifests through cellulitis or septicaemia [249]. The transition from colonization to invasive wound infection is determined by the amount, the virulence and pathogenicity of the bacteria as well as by the ability of the host to mount an effective immune defense [249]. In chronic wounds, bacteria often live in biofilms, which are communities of aggregated bacteria embedded in a self-secreted extracellular polysaccharide matrix. Biofilms protect bacteria from host immune response and antibiotic treatment [250]. Sub-infective levels of bacteria have been shown to enhance the infiltration and function of neutrophils and monocytes/macrophages, increase granulation tissue formation and angiogenesis, as a consequence, accelerating wound healing [249]. On the contrary, infection delays and impairs wound healing. In the inflammation phase, infection increases consumption of complement proteins, leading to decreased chemotaxis [251]. Bacterial products, such as short chain fatty acids from anaerobic bacteria, impair the functions of white blood cells [252]. Higher amount of cytotoxic enzymes and ROS are produced, increasing tissue damage. Bacterial exotoxins attack many types of cells and cause tissue necrosis, which is exacerbated by local hypoxemia due to vessel occlusion [253]. In the proliferation phase, bacterial metabolites inhibit keratinocyte migration and digest dermal proteins and polysaccharides, decreasing re-epithelialization [254, 255]. Infection also suppresses proliferation of fibroblasts and causes disorganized collagen production, which lead to decreased wound strength [251]. Bacterial endotoxins have been shown to reduce collagen deposition and cross-linking, which is associated with surgical dehiscence [256]. Moreover, bacterial endotoxins induce prolonged elevation of proinflammatory cytokines, e.g., IL-1β and TNF-α, which in turn increase MMP levels and decrease production of growth factors [251]. Together, infection disrupts the normal inflammation-proliferation phase transition and may cause chronicity.

### Scarring

Several lines of evidence directly link wound inflammation with the extent of scar formation. For example, the early fetal wound has minimal inflammation and exhibits scarless regeneration, whereas scar formation is exacerbated when inflammation is provoked in fetal wounds [6]. It has been shown that wounds in PU.1 null mouse, which is genetically incapable of initiating inflammatory response, heals rapidly and without scarring [257]. Moreover, oral wounds with low infiltration of inflammatory cells also heal quicker with minimal scar formation [258]. Excessive dermal fibrosis and scarring, such as hypertrophic scars and keloid, can develop after surgery, trauma, or even spontaneously in predisposed patients, which can cause physical dysfunction and psychological distress. To date, there is no satisfactory treatment available [11]. It has been shown that persistent inflammation results in up-regulation of various proinflammatory cytokines and chemokines, which further induce the expression of growth factors (e.g., PDGF, TGF-β1, activin), stimulating fibroblast proliferation, their differentiation into myofibroblasts, and production of ECM in a variety of fibrotic diseases, including hypertrophic scars and keloids [259–261]. Therefore, efficient control of the inflammatory–proliferative phase transition may be key to minimize scar formation and prevent excessive scarring.

### Age-related alterations in wound healing

With increasing age, both morphology and functions of the skin change, due to intrinsic (e.g., hormone levels) and extrinsic factors (e.g., sun exposure). The aging-related alterations in the skin result in delayed, but not defective, wound healing [262]. In the hemostasis phase, aggregation and degranulation of platelets are enhanced in the elderly [263]. In the inflammation phase, increased neutrophil response and delayed monocyte and T-cell infiltration in the wounds have been observed in the aged compared with young controls [264]. Moreover, the phagocytic activity of wound macrophages in aged mice is decreased compared with young mice, which may account for increased production of proinflammatory cytokines, including IL-1, IL-6, TNF-α, but decreased secretion of VEGF [74, 263, 265]. In the proliferation phase, diminished cell proliferation and migration, decreased cytokine production, reduced response to growth factors lead to delayed re-epithelialization, angiogenesis and granulation tissue formation in the elderly, which alterations may partially attribute to impaired response to hypoxia (reviewed in [262]). This inefficient inflammation-proliferation transition delays wound healing in old people. However, in the remodeling phase, reduced collagen turnover and increased fibroblast senescence may result in less hypertrophic and cosmetically favorable scarring in old compared with young individuals [262]. In addition to these age-related alterations in the skin, factors associated with aging, e.g., reduced sex steroid hormones, immobilization, malnutrition, medication and comorbidities (e.g., diabetes, venous insufficiency, peripheral arterial disease) render the elderly more susceptible to chronic wounds.

## Animal models of wound repair

Wound repair is a biological process conserved in all multicellular organisms. Most investigations on cell and molecular mechanisms of wound healing are performed using animal models, which are more experimentally tractable than humans. The most commonly used animal models are mice, from which we have gained significant insights on more than 100 genes important for skin wound healing over the last two decades [266]. Moreover, mice models with impaired skin wound healing, e.g., leptin receptor deficient mice (diabetes model), mice subjected to ovariectomy (skin aging model) and skin flap ischemia model, imitate some clinically relevant key aspects of chronic wounds, providing us with useful tools to explore the potential of novel therapeutic strategies. However, it is important to bear in mind the difference in wound healing between murine and human skin. For example, mouse skin does not attach to underlying tissues, therefore the initial reduction in would area is largely due to contraction of the connective tissue, whereas in human re-epithelialization plays a major role. Also mouse skin has much more hair compared with human skin, which may affect healing differently [11]. In preclinical trials of potential treatments, pig models are often used due to the similarities between pig and human skins. However, poor genetic tractability, complicated surgical procedures and high cost limit their use. Recently, Drosophila and zebrafish are used to investigate mechanisms of fundamental tissue repair [267, 268]. These models offer unique possibilities, e.g., to perform live imaging, due to their translucency, and genome-wide screen, due to their good genetic tractability and low cost.

## Concluding remarks and perspectives

Wound healing is a delicate and utterly complex process, composed of a cascade of interlocking biological events, among which the transition from inflammation to tissue regeneration may be one of the most critical and defining steps. Abnormal wound healing such as hard-to-heal ulcers and excessive scarring is associated with inefficient or failed transition between these two phases, indicating that targeting the inflammatory–proliferative phase transition could provide a new avenue for therapeutic development. It has been suggested that suppression of inflammation may be beneficial for promoting healing of chronic wounds and reducing scarring. However, it may also increase the risk of infection. Here we propose that modulation of factors that can facilitate the resolution of inflammation and initiate proliferation may be more advantageous for wound therapy.

In this review, we summarize recent research advances focusing on the mechanisms controlling the inflammatory–proliferative phase transition during skin wound healing, including cellular factors, e.g., macrophages and fibroblasts; molecular pathways, e.g., bioactive lipid mediators, reactive oxygen species and TLR signalling; gene expression regulators, e.g., transcription factors and epigenetic regulators, especially miRNAs. We propose that these factors may be promising targets for development of more effective wound therapy. However, for most of these factors we still do not know about their expression pattern and function in the situation of abnormal wound healing, which is prerequisite for designing targeted treatments. Moreover, investigation of their role in wounds with different ethiologies would be important for personalized wound treatment. Hippocrates (c 400BC) once said that ‘healing is a matter of time, but it is sometimes also a matter of opportunity’. We believe that a better understanding of mechanisms facilitating the transition from inflammation to proliferation may offer opportunities for accelerating wound healing and reducing scarring.
